# Spectroscopic Identification of Hydrogen Bond Vibrations and Quasi-Isostructural Polymorphism in N-Salicylideneaniline

**DOI:** 10.3390/molecules26165043

**Published:** 2021-08-20

**Authors:** Łukasz Hetmańczyk, Eugene A. Goremychkin, Janusz Waliszewski, Mikhail V. Vener, Paweł Lipkowski, Peter M. Tolstoy, Aleksander Filarowski

**Affiliations:** 1 Faculty of Chemistry, Jagiellonian University, 2 Gronostajowa Str., 30-387 Cracow, Poland; lukasz.hetmanczyk@uj.edu.pl; 2 Frank Laboratory of Neutron Physics, Joint Institute for Nuclear Research 6 F. Joliot-Curie str., 141980 Dubna, Russia; goremychkin@jinr.ru; 3 Faculty of Physics, University of Bialystok, 1L Ciolkowskiego str., 15-245 Bialystok, Poland; j.waliszewski@uwb.edu.pl; 4 Quantum Chemistry Department, Mendeleev University of Chemical Technology, Miusskaya Square 9, 125047 Moscow, Russia; venermv@muctr.ru; 5 Kurnakov Institute of General and Inorganic Chemistry, Russian Academy of Sciences, Leninskii Prosp. 31, 119991 Moscow, Russia; 6 Department of Physical and Quantum Chemistry, Wrocław University of Science and Technology, Wybrzeże Wyspiańnskiego 27, 50-370 Wrocław, Poland; pawel.lipkowski@pwr.edu.pl; 7 Institute of Chemistry, St. Petersburg State University, Universitetskij pr. 26, 198504 Saint Petersburg, Russia; 8 Faculty of Chemistry, University of Wrocław 14 F. Joliot-Curie str., 50-383 Wrocław, Poland

**Keywords:** *Schiff* bases, inelastic incoherent neutron scattering, hydrogen bond, isotopic effect

## Abstract

The *ortho*-hydroxy aryl *Schiff* base 2-[(E)-(phenylimino)methyl]phenol and its deutero-derivative have been studied by the inelastic incoherent neutron scattering (IINS), infrared (IR) and Raman experimental methods, as well as by Density Functional Theory (DFT) and Density-Functional Perturbation Theory (DFPT) simulations. The assignments of vibrational modes within the 3500–50 cm^−1^ spectral region made it possible to state that the strong hydrogen bond in the studied compound can be classified as the so-called quasi-aromatic bond. The isotopic substitution supplemented by the results of DFT calculations allowed us to identify vibrational bands associated with all five major hydrogen bond vibrations. Quasi-isostructural polymorphism of 2-[(E)-(phenylimino)methyl]phenol (**SA**) and 2-[(E)-(phenyl-D_5_-imino)methyl]phenol (**SA-C_6_D_5_**) has been studied by powder X-ray diffraction in the 20–320 K temperature range.

## 1. Introduction

This paper dwells on the studies of one of the most popular photo-thermochromic compounds, 2-[(E)-(phenylimino)methyl]phenol (N-salicylideneaniline), from the group of the ortho-hydroxy aryl Schiff bases. The first compounds of this type were synthesized in 1864 by Hugo Schiff [1] and have attracted attention ever since [2,3]. The ortho-hydroxy aryl Schiff bases, and materials based on them, demonstrate a number of interesting and useful characteristics. For example, they manifest polymorphic properties [4], ionic liquids’ properties [5], elastic bending capability [6] and recognised anti-cancer properties [7]. The chiral aldimines possess the photochromic feature in the crystalline state, caused by photoinduced proton transfer in the intramolecular OHN hydrogen bond [8,9]. The chiroptical and optical anisotropic properties of photomechanical Schiff bases were previously studied under UV irradiation [10]. Such properties as ferroelectricity, piezoelectricity and second-order optical non-linearity were reported [11,12].

Many of the abovementioned features are linked to the existence of an intramolecular OHN hydrogen bond and to various conformational changes that ortho-hydroxy aryl Schiff bases could undergo. The conformational changes in the excited state were first studied in the year 1962 [13] and continue to attract attention to date. The development of new synthetic routes led to a significant increase in the number of available compounds and allowed novel types of liquid crystals and photo-optical switches to be obtained [14]. Such characteristics are usually conditioned by the isomerization of the imine fragment [15,16,17,18]. Besides, isomerization of an ortho-hydroxy aryl Schiff base attached to a BODIPY chromophore was used to tune its UV absorption spectra [19].

**Figure 1:**
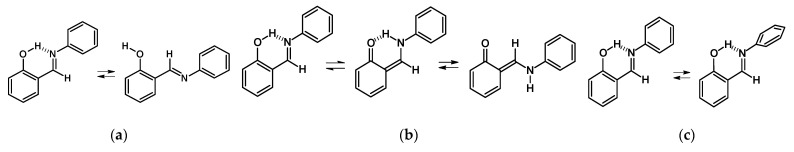
Conformational equilibria of 2-[(E)-(phenylimino)methyl]phenol.

A long discussion takes place in the literature [20,21,22,23,24,25,26,27,28,29,30] on what intramolecular changes are responsible for the emergence and fine tuning of photo-thermochromic properties of ortho-hydroxy aryl Schiff bases: isomerization of the hydroxyl- and imine- groups (Chart **a**, Figure 1) or intramolecular proton transfer with the consequent isomerization of the amino group (Chart **b**, Figure 1) or aniline ring rotation (Chart **c**, Figure 1).

The recent comprehensive studies seem to indicate that the proton transfer in the intramolecular OHN hydrogen bond and the isomerization of the aldimine fragment (Chart **b**, Figure 1) is in charge of the appearance of photo-thermochromic properties in ortho-hydroxy aryl Schiff bases [28]. Notably, the strength of the hydrogen bond and tautomeric equilibrium in it are linked to the observation of certain physical-chemical properties. In cases where the OH tautomeric form is prevailing, it is possible to observe OH isomerization, leading to the absence of photo-thermochromic properties, Chart **a**. Recently, Mielke et al. [29,30] have discovered the existence of the trans-OH form in the specific condition of matrix isolation. However, the prevailing of the NH tautomeric form makes it possible to observe the isomerization of the amino group (Chart **b**) and, therefore, the emergence of photo-thermochromic properties. It is important to underline the contribution by Ogawa et al. [24] who were the first to show the existence of trans-NH form in the solid state by X-ray method. The possibility of rotation of aldimine ring (Chart **c**) was first demonstrated by Cohen et al. [13,20], and later it was associated with the appearance of polymorphism of the studied compound.

**Figure 2:**
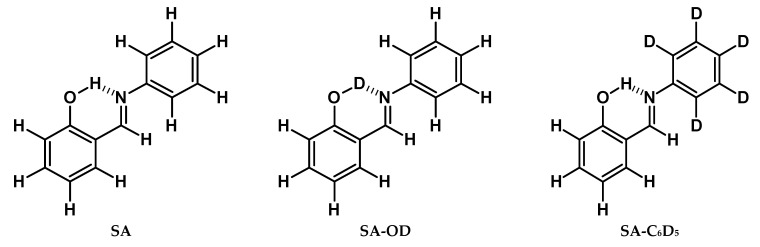
Structures of studied 2-[(E)-(phenylimino)methyl]phenol and its isotopologues.

The conformational changes, occurring in ortho-hydroxy aryl Schiff bases in different conditions and states, were investigated mostly by the methods of vibrational spectroscopy in the middle spectral range [29,30,31,32,33,34,35,36], while the low- and high-frequency hydrogen bridge vibrations have never been in the focus of the spectroscopic studies before. Therefore, in order to fill in the gap, we have synthesized 2-[(E)-(phenylimino)methyl]phenol (N-salicylideneaniline, **SA**) and its deutero-derivatives (**SA-OD** and **SA-C_6_D_5_**, see Figure 2) to analyse the vibrational spectra measured by IINS, Raman and IR methods. The quantum-mechanical DFT and DFPT calculations, as well as Potential Energy Distribution (PED) analysis, have been conducted to assign the spectral bands. The isotopic replacements of the bridged hydrogen for deuterium (O-H···N → O-D···N) and the hydrogen atoms for the deuterium atoms in aldimine ring (NC_6_H_5_ → NC_6_D_5_) were performed to additionally confirm the assignment. To that end, the Isotopic Spectral Ratios (ISRs) were used, which are defined for a given vibrational mode as the ratio of frequencies for non-deuterated and deuterated species. The ISR values are well studied for high-frequency stretching and bending proton vibrations [37], but poorly studied for the low-frequency hydrogen bridge vibrations.

Moreover, this paper delves into the spectroscopic manifestations of polymorphism of N-salicylideneaniline and its isotopologues in a wider temperature range in 20–320 K. N-salicylideneaniline was studied earlier in papers [27,28] in the 100–320 K temperature range.

## 2. Results and Discussion

### 2.1. Assignment of Hydrogen Bonding Vibrations

**Scheme 1:**
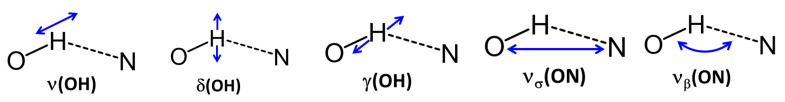
The OHN hydrogen bond vibrations of the studied 2-[(E)-(phenylimino)methyl]phenols.

This part of the paper focuses on the assignments of the spectral bands to the vibrations involving the hydroxyl group (ν(OH), δ(OH) and γ(OH))) and the hydrogen bridge (ν_σ_(ON) and ν_β_(ON)), Scheme 1, continuing and expanding our studies started in Ref. [38].

We start the discussion with the high-frequency region of vibrational spectra, which are presented in Figure S1 in the supporting information for brevity. The broad band located within the range of 2900–1500 cm^−1^ in the measured infrared spectrum is assigned to the stretching vibrations ν(OH) as a result of the comparison of the spectra of the non-deuterated and deuterated isopologues (ISR = 1.320). This band is characteristic for OH form of Schiff bases (enol-imine) and not to NH form (keto-amine). The ν(OH) band is rather wide and can be assigned to the so-called Zundel’s continuum absorption [39]. This fact proves that the hydrogen bond in these compounds is strong. Moreover, the intensity of the ν(OH) band is rather weak despite its noticeable shift towards lower frequencies, which indicates that the hydrogen bond in **SA** is a quasi-aromatic one, which is also referred to as resonance-assisted hydrogen bond (RAHB) [40,41,42,43,44,45,46,47,48].

**Figure 3:**
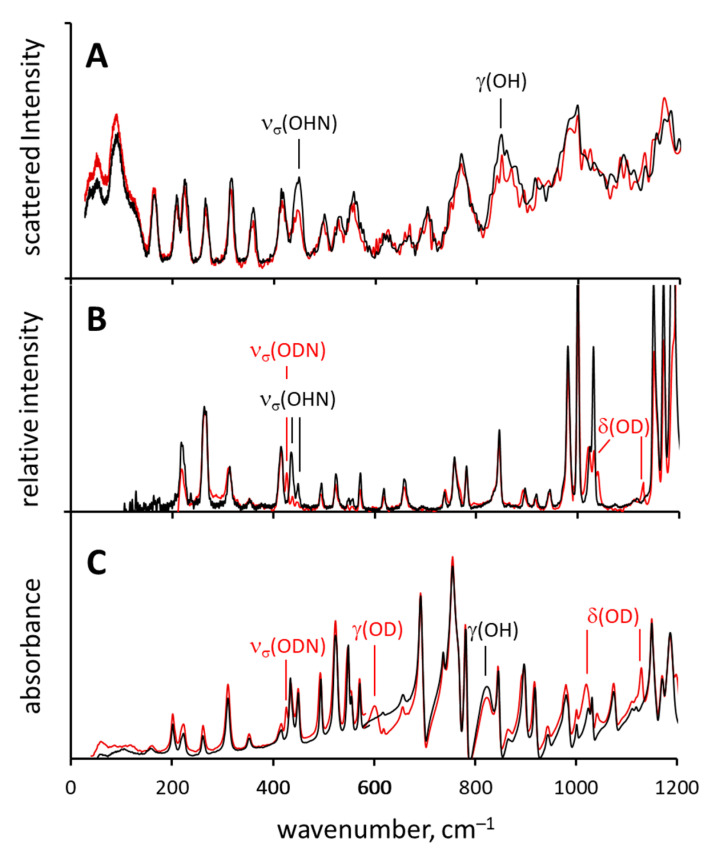
Normalized experimental IINS (**A**), Raman (**B**) and IR (**C**) spectra of **SA** (black line) and its deuterated derivative **SA-OD** (red lines).

The low-frequency region of the IR, Raman and IINS spectra of non-deuterated (**SA**) and deuterated (**SA-OD**) compounds are shown in Figure 3. The experimental spectra were interpreted using the calculated vibrational spectra (DFT) and the results of the PED analysis (Tables S2–S4).

When it comes to the in-plane deformational vibrations δ(OH), they are not characteristic for ortho-hydroxy Schiff bases [33,35,38]. According to PED analysis, δ(OH) vibration is mixed with the ν(C_alk_C_alk_) and γ(CH) vibrations. After the OH → OD substitution the bands located at 1571 and 1484 cm^−1^ (both IR and Raman active) decrease in intensity and shift slightly, while the more characteristic δ(OD) bands appear at ca. 1130 cm^−1^ and ca. 1020 cm^−1^ both in IR and Raman spectra of **SA-OD** (Figure 3).

As for the out-of-plane deformational vibration γ(OH), in the IR spectrum of **SA** the corresponding band is found in the range of 860–820 cm^−1^, but it strongly overlaps with the bands of other vibrations and was identified with the help of DFT calculations. However, in the IR spectra of **SA-OD** this band is clearly visible at 601 cm^−1^. The γ(OH) vibration of **SA** is practically Raman-inactive, as in the 860–820 cm^−1^ region of the Raman spectrum there are no clearly identifiable bands which would be sensitive to the OH → OD replacement. The band at 848 cm^−1^ in the IINS spectrum drops in intensity after the deuteration and thus it was assigned to the γ(OH) vibration. Note, that for IINS method the scattering cross-section for the deutron is much smaller than that of the proton and thus the contribution of the vibrations involving deutrons in IINS spectra is weak. The experimentally observed positions of γ(OH) bands and the H/D isotope effects on them are consistent with the literature data [49,50,51].

**Figure 4:**
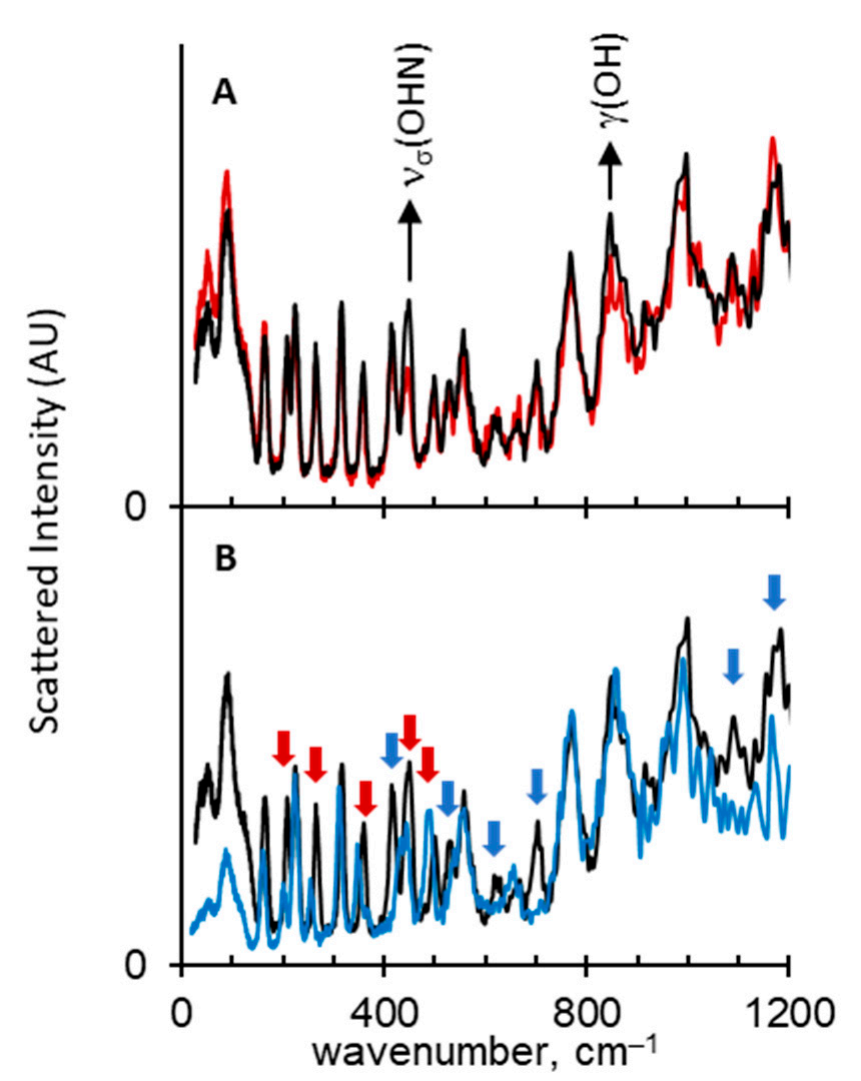
Normalized IINS spectra of **SA** (black traces), **SA-OD** (panel (**A**), red trace) and **SA-C_6_D_5_** (panel (**B**), blue trace). Blue arrows: bands of γ(CH) and τ(CH) vibrations, which disappear after deuteration. Red arrows: bands of various vibrations, which shift after the deuteration. See text for more details.

The stretching vibration of the hydrogen bridge (ν_σ_(OHN)) gives rise to the isotope-sensitive bands at 449 cm^−1^ in the IINS spectrum and two bands at 448 and 434 cm^−1^ in the Raman spectrum of **SA** (Figure 3). The intensity of these bands is greatly decreased in the IINS and the Raman spectra of **SA-OD**, while a new band appears at 425 cm^−1^ in the Raman spectrum, assigned to the vibration of the ODN bridge, ν_σ_(ODN). The IINS spectrum does not show the band of the deformational vibration ν_β_(OHN) due to insignificant deformational motion of the bridged proton. This phenomenon was described earlier in Ref. [38]. To make the interpretation of the hydrogen bridge vibrations more accurate and reliable, the synthesis of **SA-C_6_D_5_** isotopologue was performed (deuteration in the aldimine ring, NC_6_H_5_ → NC_6_D_5_). In Figure 4 the IINS spectra of **SA**, **SA-OD** and **SA-C_6_D_5_** are compared. Upon deuteration in the hydrogen bridge site (**SA-OD**), the intensity of the bands assigned to the γ(OH) and ν_σ_(OHN) vibrations (848 cm^−1^ and 449 cm^−1^, respectively) is decreasing (Figure 4A), while the IINS spectrum of the **SA-C_6_D_5_** derivative features a more complicated picture (Figure 4B). Firstly, the IINS spectrum of **SA-C_6_D_5_** is characterized by the disappearance of a number of bands assigned to γ(CH) and τ(CH) vibrations, which were located at 1183, 1175, 1153, 1089, 703 and 521 cm^−1^ in the spectrum of **SA** (see blue arrows in Figure 4). Secondly, the bands visible at 208, 264, 359 and 495 cm^−1^ in the spectrum of **SA** (see red arrows in Figure 4B) shift to lower frequencies, namely, to 202, 255, 345 and 491 cm^−1^, respectively. The intensity of the first three bands is decreasing, while that of the fourth one is increasing slightly. Judging from Tables S2 and S4, these bands stem from the skeleton vibrations of the aldimine ring, where the deuteration occurs.

The band at 449 cm^−1^, which is assigned to ν_σ_(OHN) vibration (also marked by the red arrow in Figure 4B), is also sensitive to deuteration in the aldimine ring: the band broadens and its peak intensity decreases. Such a long-range H/D isotope effect on hydrogen bond vibrations is reported here for the first time. Below in Section 2.3 it is shown that **SA** exists as a mixture of two quasi-isostructural polymorphs and the shift of the ν_σ_(OHN) band might be associated with the change of the mole fractions of these polymorphs upon **SA** → **SA-C_6_D_5_** deuteration, though this question requires additional consideration which is beyond the scope of this work.

### 2.2. Spectral Manifestations of Polymorphism

**Figure 5:**
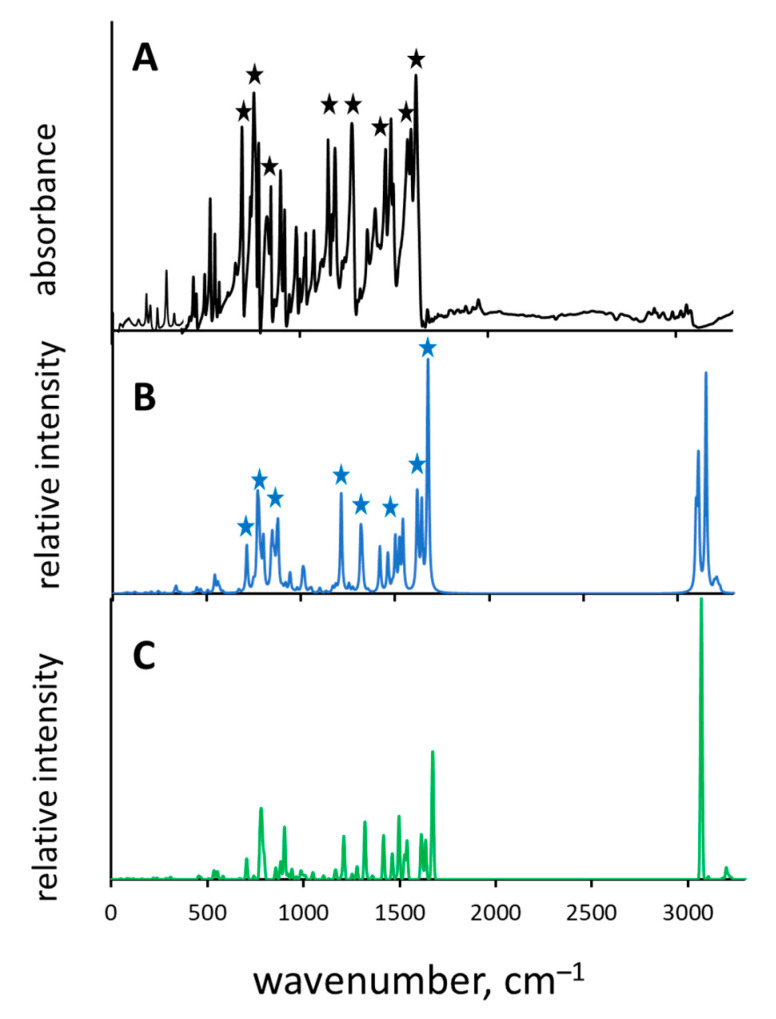
Normalized IR experimental ((**A**), black trace; measured at 295 K) and calculated by DFPT method spectra of polymorphs α_2_ ((**B**), blue trace) and β ((**C**), green trace) of **SA**. Black and blue asterisks: the most informative bands, allowing one to identify polymorph α_2_. See text for more details.

The **SA** could crystallize in three polymorphic states—called α_1_, α_2_ and β—the crystal structures and cell packing of which were earlier published by F. Arod in papers [27,28]; for a visual representation of the cell packing see Figure 2 (polymorph β) and Figure 8 (polymorphs α_1_ and α_2_) in [27]. All three polymorphs exhibit enol-imine form with intramolecular OH···N hydrogen bond. These polymorphs differ only slightly in molecular positions and conformations, representing “very close points in the crystal structure landscape” [52,53,54]; one of the larger differences between α and β states is the rotational conformation of the aldimine ring (Chart **c**, Figure 1). The polymorphs α_1_ and α_2_ are called quasi-isostructural. The structural mobility and polymorphism of different compounds [55,56,57,58,59], and Schiff bases in that number [60,61], was investigated by various methods. In this part of the paper our goal was to determine if vibrational spectra could be used to unambiguously identify the particular polymorphic state and which vibrational marker modes would be most informative. For that purpose, the crystallographic structures of polymorphs α_2_ and β were optimized with CRYSTAL software, the IR spectra were calculated by DFPT method and compared with the experimental one. The result of the comparison is shown in Figure 5. There is a reasonably good agreement—both in bands positions and their relative intensities—between the experimental spectrum (Figure 5A) obtained at 295 K in which polymorph α_2_ prevails and the calculated spectrum of polymorph α_2_ (Figure 5B), while the agreement with that of polymorph β is significantly worse (Figure 5C). The most informative bands are marked by asterisks in Figure 5A,B. These bands are assigned to the following vibrations: ν(C=N), ν(C_ar_C_ar_), γ(CH), τ(CH) and τ(CC).

The abovementioned observations support the applicability of DFPT computational method for the research of polymorphic states.

### 2.3. X-ray Powder Diffraction (XPD) Study of Polymorphism in SA

**Figure 6:**
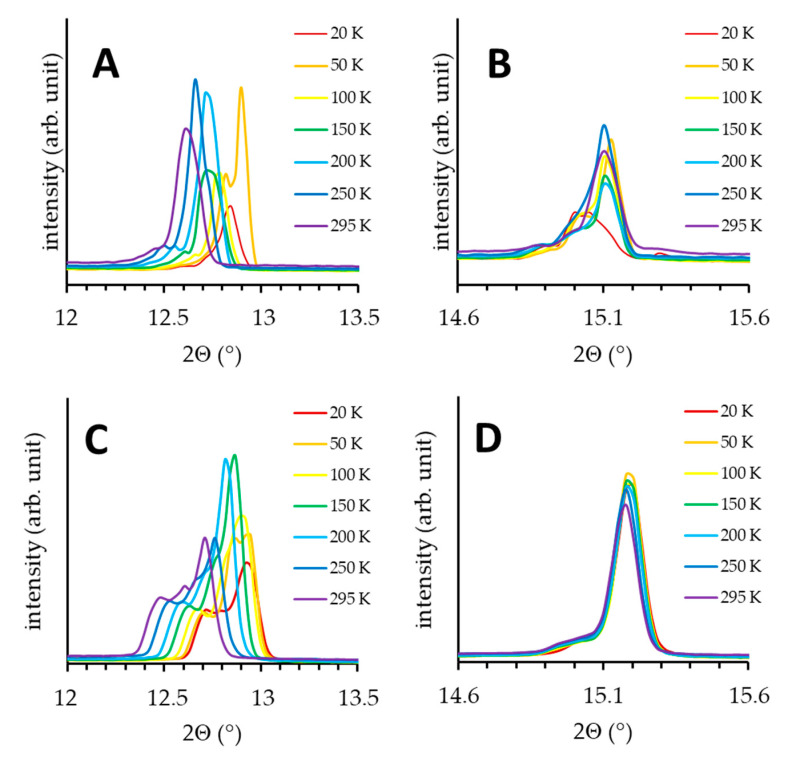
Temperature behaviour of (002) (**A**,**C**) and (0-11) (**B**,**D**) reflections of **SA** top (**A**,**B**) and **SA-C_6_D_5_** bottom (**C**,**D**) panels.

X-ray Powder Diffraction measurements of **SA** and **SA-C_6_D_5_** were carried out in the 20–320 K temperature range. The X-ray diffraction pattern for **SA-OD** is not discussed here, because the results closely match those for **SA**. The experiments revealed that both **SA** and **SA-C_6_D_5_** crystallize in a triclinic form, which is in agreement with the single crystal X-ray data for polymorph α_1_ obtained earlier [27]. For **SA**, several reflexes are observed as dual signals in the 20–295 K temperature range. As an example, in Figure 6 the reflexes, 002 and 0-11, for **SA** and **SA-C_6_D_5_** are shown. The positions and relative intensities of components of the dual signals are temperature dependent. Similar observations are valid for the deuterated derivative **SA-C_6_D_5_** (Figure 6C,D). Such behaviour is often attributed to the co-existence of two quasi-isostructural polymorphs, preserving the same crystal symmetry [52,53,54,55,56]. In case of **SA**, following the results of Refs. [27,28] we assign these polymorphs to α_1_ and α_2_ forms.

**Figure 7:**
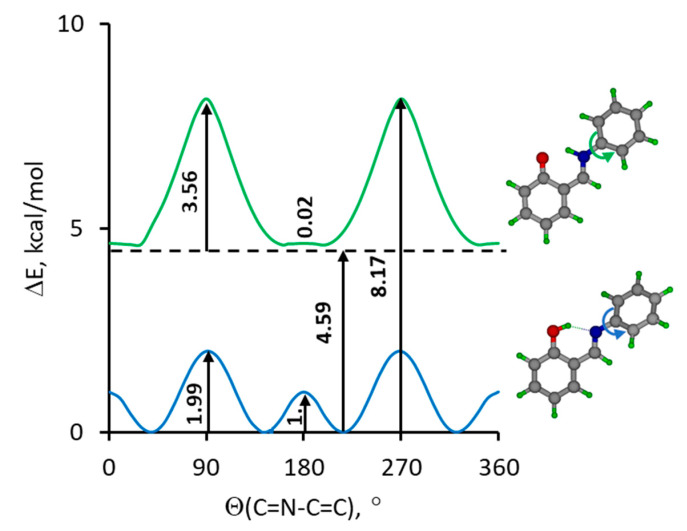
The calculated potential energy profile (B3LYP/6-311++G(d,p)) for a gradual rotation around the N-C bond for enol-imine (blue line) and keto-amine (green line) forms of studied Schiff base.

For a better understanding of this phenomenon we performed DFT calculations (in the gas phase) of the potential curves for the rotation of the aldimine fragment of **SA** in the enol-imine and keto-amine forms. The calculation confirmed that the structure of the keto-amine form is evidently flat and the twist of the aldimine fragment by up to 20° virtually does not change the potential energy (Figure 7, top). In contrast, the optimized geometry of the enol-imine form is not flat: torsional angle Θ(C=N-C=C) = 40° (Figure 7, bottom) and crossing of the phenol ring plane requires to overcome a 1 kcal/mol barrier. According to the postulate of Benstein et al. [62], such a barrier in a non-homogeneous environment of a crystal lattice could make it possible to obtain two polymorphic forms. Though the keto-amine form is less stable than the enol-imine form by ca. 4.6 kcal/mol and unlikely to be present at room temperature, one could speculate see that the flat structure of the keto-amine form would not be prone to polymorphism.

The keto-amine if the keto-amine form would be,

Upon further heating, a significant change of diffractograms of **SA** and **SA-C_6_D_5_** is observed at ca. 310 K (see the set of diffractograms in Figures S2 and S3). Based on the available XPD data, it is difficult to speculate which structural changes are responsible for this, but it is unlikely to be the α ⟷ β transition, because the melting temperature of studied crystal was 325 K, coinciding with that previously reported for α_1_ in Ref. [27].

## 3. Materials and Methods

### 3.1. Compounds and Deuteration

2-[(E)-(phenylimino)methyl]phenol (**SA**) and 2-[(E)-(phenyl-D_5_-imino)methyl]phenol (**SA-C_6_D_5_**) were synthesized from stoichiometric mixtures of the salicylaldehyde with aniline or aniline-D_5_ in refluxing methanol, respectively. The solvents were purchased from *Sigma-Aldrich* and used without further purification. For the deuteration in the mobile proton site, the solution of 2-[(E)-(phenylimino)methyl]phenol in methanol-OD was heated to 60 °C and refluxed during 30 min, then the methanol was removed by evaporation, leaving **SA-OD**. The deuteration degree was estimated to be ca. 90%.

### 3.2. Infrared, Raman and IINS Measurements

The infrared measurements were performed using a *Bruker Vertex 70v* spectrometer. The spectra were collected with a resolution of 2 cm^−1^. The FT-FIR spectra (500–50 cm^−1^) were collected for sample suspended in Apiezon N grease and placed on a polyethylene (PE) disc. The FT-MIR spectra were collected for sample in a KBr pellet. The Raman spectra of the analysed samples were obtained using an FT-Nicolet Magma 860 spectrophotometer. The In:Ga:Ar laser excitation at 1064 nm was employed for the Raman measurements. The spectra were recorded at the room temperature with the spectral resolution 4 cm^−1^ and with 512 scans. Neutron scattering data were collected at the pulsed IBR-2 reactor in the Joint Institute of Nuclear Research (Dubna, Russia) using the time-of-flight inverted geometry spectrometer NERA at 10 K temperature. The experimental features are described in Ref. [50].

### 3.3. X-ray Powder Diffraction

The powder X-ray measurements were performed using EMPYREAN equipped with the water-cooled X-ray tube with Cu anode with the wavelengths of characteristic radiation λ_CuKα1_ = 1.54056 Å and λ_CuKα2_ = 1.54443 Å. In order to suppress the K_β_ radiation the Ni filter was used. The central part of the data acquisition system was the silicon strip detector PIXcel. The low temperature measurements were made using the closed cycle helium cryostat Phenix—Oxford Cryosystems.

### 3.4. Calculations

Quantum-mechanical calculations using the B3LYP functional (DFT, the three-parameter exchange hybrid functional of Becke [63], and gradient-corrected correlation functional of Lee, Yang and Parr) [64] with the 6-311++G(d,p) basis set [65,66,67] were performed for full geometry optimizations with the *Gaussian* 16 Rev. C01 suite of program [68]. The structures were visualized by the MOLDEN program [69]. The potential energy distribution (PED) of the normal modes was calculated in terms of natural internal coordinates using the Gar2ped program [70]. Static periodic (solid-state) DFT calculations (DFPT *Density-Functional Perturbation Theory*) were performed in the CRYSTAL09 [71,72] software package using the B3LYP functional with the Grimme D2 dispersion correction [73] and 6-31G** basis set [67]. The space groups and unit cell parameters of the considered crystals obtained in the experimental studies [27,28] were fixed and structural relaxations were limited to the position of atoms. Such approximation gives a reasonable description of the structure and spectroscopic features of intra- and intermolecular H-bonds of different types and strengths in molecular crystals [74,75,76]. The experimental atomic positions were used as the starting point in the periodic DFT computations. Periodic DFT computations were conducted for polymorphs α_2_ and β. The disordered α_1_-polymorph [27] is not suitable for periodic calculations. Since α_1_ and α_2_ polymorphs are quasi-isostructural, we assumed that their IR spectra are very close to each other. Therefore, only the IR spectrum of the second polymorph was considered in the present study. Periodic DFT computations of α_2_-polymorph led to the appearance of imaginary frequencies. This problem is usually solved by reducing the space symmetry of a crystal [77,78]. Reducing the space symmetry group to P1 allowed us to get rid of imaginary frequencies of α_2_-polymorph. An accurate interpretation of the experimental spectrum assumes knowledge of the relative stability of polymorphs α_2_ and β. This requires the calculation of the sublimation enthalpy, which is very cumbersome in the case of conformationally mobile molecules [79]. In addition, the error in the calculated values can reach 15 kJ/mol [80]. Such an accuracy of calculations is hardly suitable for describing “very close points in the crystal structure landscape” of crystals with conformationally mobile molecules, e.g., see [81].

## 4. Conclusions

The paper presents the synthesis of 2-[(E)-(phenylimino)methyl]phenol (**SA**) and its two deutero-derivatives (**SA-OD** and **SA-C_6_D_5_**). The bands corresponding to hydrogen bond vibrational modes (see Scheme 1) were assigned in experimental IR, IINS and Raman spectra. The analysis of the obtained spectral results proved that in a crystal state, **SA** exists in enol-imine form with a so-called quasi-aromatic intramolecular OH···N hydrogen bond of medium strength. The measured IINS spectra **SA** and **SA-C_6_D_5_** made it possible to show the influence of deuteron replacement in the remote group (aldimine ring) on the hydrogen bridge stretching vibrations ν_σ_(OHN). The calculations of structure and vibrational spectra of two polymorphs (earlier studied by X-ray method [27]) performed by static periodic (solid state) DFT method showed a good agreement between the measured and calculated spectra, allowing for spectroscopic distinction between polymorphs α_2_ and β, as well as confirming the applicability of DFPT calculations for characterization of polymorphic states. Based on analysis of IR spectra, the **SA** in the studied samples was shown to exist in α-form. However, the XPD measurements carried out for **SA** and **SA-C_6_D_5_** showed dual signals for several reflexes and did not show a phase transition in the temperature range from 20 to 295 K. On the basis of these measurements two quasi-isostructural polymorphs of **SA** (most probably, so-called α_1_ and α_2_ forms) were assumed to co-exist in the mentioned temperature range.

## Data Availability

All data are available within the article or Supplementary Materials.

## Supplementary Materials

The following are available online, Figure S1: Normalized experimental Raman and IR spectra of **SA** and its deuterated derivative **SA-OD**. Figure S2: Temperature behaviour of XRD patterns for **SA** and **SA-C_6_D_5_**. Figure S3: Temperature dependence of X-ray powder diffraction patterns of **SA** and **SA-C****6****D****5.** Table S1: Definitions of the internal coordinates used in the potential energy distribution (PED) analysis for the assignments of the vibrational spectra, Table S2: Experimental IR, Raman, IINS and calculated (B3LYP/6-311+G(d,p)) spectral data of **SA**, Table S3: Experimental IR, Raman and calculated (B3LYP/6-311+G(d,p)) spectral data of **SA-OD**, Table S4: Experimental IINS and calculated (B3LYP/6-311+G(d,p)) spectral data of **SA-C_6_D_5_** for the region 0–1200 cm^–1^.

## Sample Availability

Samples of the studied compounds are not available from the authors.

## References

[1] Schiff H. (1864). Mittheilungen aus dem Universitätslaboratorium in Pisa Eine neue Reihe organischer Basen. Justus Liebigs Ann. Chem..

[2] Tidwell T.T. (2008). Hugo (Ugo) Schiff, Schiff Bases, and a Century of β-Lactam Synthesis. Angew. Chem..

[3] Anselrnino O. (1905). Isomere Schiff’sche Basen. Ber. Dtsch. Chem. Ges..

[4] Carletta A., Dubois J., Tilborg A., Wouters J. (2015). Solid-state investigation on a new dimorphic substituted *N*-salicylidene compound: Insights into its thermochromic behaviour. CrystEngComm.

[5] Ossowicz P., Janus E., Szady-Chelmieniecka A., Rozwadowski Z. (2016). Influence of modification of the amino acids ionic liquids on their physico-chemical properties: Ionic liquids versus ionic liquids-supported Schiff bases. J. Mol. Liq..

[6] Liu H., Ye K., Zhang Z., Zhang H. (2019). An Organic Crystal with High Elasticity at an Ultra-Low Temperature (77 K) and Shape ability at High Temperatures. Angew. Chem. Int. Ed..

[7] Napier I., Ponka P., Richardson D.R. (2005). Iron trafficking in the mitochondrion: Novel pathways revealed by disease. Blood.

[8] Kawato T., Koyama H., Kanatomi H., Shigemizu H. (1997). Difference in the Rate of Photo-induced Unimolecular Motion of Chiral Salicylideneamines in the Chiral Crystal Environments. Chem. Lett..

[9] Koshima H., Matsuo R., Matsudomi M., Uemura Y., Shiro M. (2013). Light-Driven Bending Crystals of Salicylidenephenylethylamines in Enantiomeric and Racemate Forms. Cryst. Growth Des..

[10] Takanabe A., Tanaka M., Johmoto K., Uekusa H., Mori T., Koshima H., Asahi T. (2016). Optical Activity and Optical Anisotropy in Photomechanical Crystals of Chiral Salicylidenephenylethylamines. J. Am. Chem. Soc..

[11] Centore R., Jazbinsek M., Tuzi A., Roviello A., Capobianco A., Peluso A. (2012). A series of compounds forming polar crystals and showing single-crystal-to-single-crystal transitions between polar phases. CrystEngComm.

[12] Takanabe A., Katsufuji T., Johmoto K., Uekusa H., Shiro M., Koshima H., Asahi T. (2017). Reversible Single-Crystal-to-Single-Crystal Phase Transition of Chiral Salicylidenephenylethylamine. Crystals.

[13] Cohen M.D., Schmidt G.M. (1962). Photochromy and thermochromy of anils. J. Phys. Chem..

[14] Riddle J.A., Bollinger J.C., Lee D. (2005). Escape from a Nonporous Solid: Mechanically Coupled Biconcave Molecules. Angew. Chem. Int. Ed..

[15] Jia Y., Li J. (2015). Molecular Assembly of Schiff Base Interactions: Construction and Application. Chem. Rev..

[16] Kandappa S.K., Valloli L.K., Ahuja S., Parthiban J., Sivaguru J. (2021). Taming the excited state reactivity of imines—from non-radiative decay to aza Paternó–Büchi reaction. Chem. Soc. Rev..

[17] Muriel W.A., Botero-Cadavid J.F., Cardenas C., Rodriguez-Cordoba W. (2018). A theoretical study of the photodynamics of salicylidene-2-anthrylamine in acetonitrile solution. Phys. Chem. Chem. Phys..

[18] Matamoros E., Cintas P., Light M.E., Palacios J.C. (2019). Electronic effects in tautomeric equilibria: The case of chiral imines from d-glucamine and 2-hydroxyacetophenones. Org. Biomol. Chem..

[19] Filarowski A., Lopatkova M., Lipkowski P., Van der Auwerar M., Leen V., Dehaen W. (2015). Solvatochromism of BODIPY-Schiff base Dye. J. Phys. Chem. B.

[20] Cohen M.D., Schmidt G.M.J., Flavian S. (1964). Topochemistry. Part VI. Experiments on photochromy and thermochromy of crystalline anils of salicylaldehydes. J. Chem. Soc..

[21] Andes R.V., Manikowski D.M. (1968). Photochromism of Salicylidene Aniline. Appl. Opt..

[22] Destro R., Gavezzotti A., Simonetta M. (1978). Salicylideneaniline. Acta Crystallogr..

[23] Hadjoudis E., Vittorakis M., Moustakali-Mavridis I. (1987). Photochromism of organic compounds in the crystal state. Tetrahedron.

[24] Ogawa K., Kasahara Y., Ohtani Y., Harada J. (1998). Crystal Structure Change for the Thermochromy of *N*-Salicylideneanilines. The First Observation by X-ray Diffraction. J. Am. Chem. Soc..

[25] Gao A.-H., Wang M.-S. (2017). Nonadiabatic *ab initio* molecular dynamics study of photoisomerization in *N*-salicilydenemethylfurylamine (SMFA). J. Chem. Phys..

[26] Rawat M.S.M., Mal S., Singh P. (2015). Photochromism in Anils—A Review. Open Chem. J..

[27] Arod F., Pattison P., Schenk K.J., Chapuis G. (2007). Polymorphism in *N*-Salicylideneaniline Reconsidered. Cryst. Growth Des..

[28] Arod F., Gardon M., Pattison P., Chapuis G. (2005). The α2-polymorph of salicylideneaniline. Acta Crystallogr..

[29] Grzegorzek J., Filarowski A., Mielke Z. (2011). The photoinduced isomerization and its implication in the photo-dynamical processes in two simple Schiff bases isolated in solid argon. Phys. Chem. Chem. Phys..

[30] Grzegorzek J., Mielke Z., Filarowski A. (2010). C=N–N=C conformational isomers of 2’-hydroxyacetophenone azine: FTIR matrix isolation and DFT study. J. Mol. Struct..

[31] Sıdır İ., Gülseven Sıdır Y., Góbi S., Berber H., Fausto R. (2021). Structural Relevance of Intramolecular H-Bonding in *Ortho*-Hydroxyaryl Schiff Bases: The Case of 3-(5-bromo-2-hydroxybenzylideneamino) Phenol. Molecules.

[32] Majerz I., Pawlukojć A., Sobczyk L., Dziembowska T., Grech E., Szady-Chełmieniecka A. (2000). The infrared, Raman and inelastic neutron scattering studies on 5-nitro-N-salicylideneethylamine. J. Mol. Struct..

[33] Filarowski A., Koll A., Lipkowski P., Pawlukojć A. (2008). Inelastic neutron scattering and vibrational spectra of 2-(*N*-methyl-α-iminoethyl)-phenol and 2-(*N*-methyliminoethyl)-phenol: Experimental and theoretical approach. J. Mol. Struct..

[34] Moosavi-Tekyeh Z., Dastani N. (2015). Intramolecular hydrogen bonding in N-salicylideneaniline: FT-IR spectrum and quantum chemical calculations. J. Mol. Struct..

[35] Pająk J., Maes G., De Borggraeve W.M., Boens N., Filarowski A. (2007). Matrix-isolation FT-IR and theoretical investigation of the vibrational properties of the sterically hindered *ortho*-hydroxy acylaromatic *Schiff* bases. J. Mol. Struct..

[36] Dziembowska T., Szafran M., Katrusiak A., Rozwadowski Z. (2009). Crystal structure of and solvent effect on tautomeric equilibrium in Schiff base derived from 2-hydroxy-1-naphthaldehyde and methylamine studied by X-ray diffraction, DFT, NMR and IR methods. J. Mol. Struct..

[37] Sobczyk L., Obrzud M., Filarowski A. (2013). H/D Isotope Effects in Hydrogen Bonded Systems. Molecules.

[38] Kwocz A., Panek J.J., Jezierska A., Hetmańczyk Ł., Pawlukojć A., Kochel A., Lipkowski P., Filarowski A. (2018). A molecular roundabout: Triple cycle-arranged hydrogen bonds in light of experiment and theory. New J. Chem..

[39] Zundel G., Schuster P., Zundel G., Sandorfy C. (1976). Easily Polarizable Hydrogen Bonds—Their Interactions with the Environment—IR Continuum and Anomalous Large Conductivity. The Hydrogen Bond: Recent Developments in Theory and Experiments.

[40] Shigorin D.N., Sokolov N.D., Chulanovsky A.D. (1964). Vodorodnaya Svyaz’ (Hydrogen Bond).

[41] Hadži D., Bratos S., Schuster P., Zundel G., Sandorfy C. (1976). Vibrational spectroscopy of the hydrogen bond. The Hydrogen Bond: Recent Developments in Theory and Experiments.

[42] Tsubomura H. (1956). Nature of the Hydrogen Bond. III. The Measurement of the Infrared Absorption Intensities of Free and Hydrogen-Bonded OH Bands. Theory of the Increase of the Intensity Due to the Hydrogen Bond. J. Chem. Soc..

[43] Hadzi D. (1956). Absorption spectra and structure of some solid hydroxyazo-compounds. J. Chem. Soc..

[44] Filarowski A., Koll A. (1996). Intergrated intensity of ν_s_(OH) absorption bands in bent hydrogen bonds in *ortho*-dialkylaminomethyl phenols. Vib. Spectrosc..

[45] Filarowski A., Koll A. (1998). Specificity of the intramolecular hydrogen bond. The differences in spectroscopic characteristics of the intermolecular and intramolecular H-bonds. Vib. Spectrosc..

[46] Takasuka M., Matsui Y. (1979). Experimental Observations and CN D0/2 Calculations for Hydroxy Stretching Frequency Shifts, Intensities, and Hydrogen Bond Energies of Intramolecular Hydrogen Bonds in ortho-Substituted Phenols. J. Chem. Soc. Perkin II.

[47] Nguyen Y.H., Lampkin B.J., Venkatesh A., Ellern A., Rossini A.J., VanVeller B. (2018). Open-Resonance-Assisted Hydrogen Bonds and Competing Quasiaromaticity. J. Org. Chem..

[48] Gilli G., Gilli P. (2009). The Nature of the Hydrogen Bond: Outline of a Comprehensive Hydrogen Bond Theory.

[49] Hetmańczyk Ł., Szklarz P., Kwocz A., Wierzejewska M., Pagacz-Kostrzewa M., Melnikov M.Y., Tolstoy P.M., Filarowski A. (2021). Polymorphism and Conformational Equilibrium of Nitro-Acetophenone in Solid State and under Matrix Conditions. Molecules.

[50] Piękoś P., Jezierska A., Panek J.J., Goremychkin E.A., Pozharskii A.F., Antonov A.S., Tolstoy P.M., Filarowski A. (2020). Symmetry/asymmetry of the NHN hydrogen bond in protonated 1,8-bis(dimethylamino)naphthalene. Symmetry.

[51] Jóźwiak K., Jezierska A., Panek J.J., Goremychkin E.A., Tolstoy P.M., Shenderovich I.G., Filarowski A. (2020). Inter- *vs*. intra-molecular hydrogen bond patterns and proton dynamics in phthalic acid associates. Molecules.

[52] Prohens R., Barbas R., Font-Bardia M. (2020). Morphotropism and “Quasi-Isostructurality” in the Three High Z′ Concomitant Polymorphs of Efinaconazole. Cryst. Grow. Des..

[53] Kálmán A., Párkányi L., Argay G. (1993). Classification of the isostructurality of organic molecules in the crystalline state. Acta Cryst..

[54] Dey D., Thomas S.P., Spackman M.A., Chopra D. (2016). Quasi-isostructural polymorphism’ in molecular crystals: Inputs from interaction hierarchy and energy frameworks. Chem. Commun..

[55] Fischer J., Lima J.A., Freire P.T.C., Melo F.E.A., Havenith R.W.A., Filho J.M., Broer R., Eckert J., Bordallo H.N. (2013). Molecular flexibility and structural instabilities in crystalline L-methionine. Biophys. Chem..

[56] De Souza J.M., Freire P.T.C., Argyriou D.N., Stride J.A., Barthòs M., Kalceff W., Bordallo H.N. (2009). Raman and Neutron Scattering Study of Partially Deuterated l-Alanine: Evidence of a Solid-Solid Phase Transition. ChemPhysChem.

[57] Hetmańczyk J., Hetmańczyk Ł., Bilski P., Kozak A. (2020). Complex water dynamics in crystalline [Ca(H2O)2](ReO4)2, studied by the vibrational spectroscopy and proton magnetic resonance. J. Mol. Struct..

[58] Szostak E., Hetmańczyk J., Migdał-Mikuli A. (2015). Inelastic and elastic neutron scattering studies of the vibrational and reorientational dynamics, crystal structure and solid–solid phase transition in [Mn(OS(CH3)2)6](ClO4)2 supported by theoretical (DFT) calculations. Spectrochim. Acta A.

[59] Hetmańczyk J., Hetmańczyk Ł., Nowicka-Scheibe J., Pawlukojć A., Maurin J.K., Schilf W. (2021). Structural, Thermal, and Vibrational Properties of N,N-Dimethylglycine–Chloranilic Acid—A New Co-Crystal Based on an Aliphatic Amino Acid. Materials.

[60] Shapenova D.S., Shiryaev A.A., Bolte M., Kukułka M., Szczepanik D.W., Hooper J., Babashkina M.G., Mahmoudi G., Mitoraj M.P., Safin D.A. (2020). Resonance Assisted Hydrogen Bonding Phenomenon Unveiled from Both Experiment and Theory—An Example of New Family of Ethyl N-salicylideneglycinate Dyes. Chem. Eur. J..

[61] Safin D.A., Robeyns K., Babashkina M.G., Filinchuk Y., Rotaru A., Jureschi C., Mitoraj M.P., Hooper J., Brela M., Garcia Y. (2016). Polymorphism driven optical properties of an anil dye. CrystEngComm.

[62] Cruz-Cabeza A.J., Bernstein J. (2014). Conformational Polymorphism. Chem. Rev..

[63] Becke A.D. (1993). Density-functional thermochemistry. III. The role of exact exchange. J. Chem. Phys..

[64] Lee C., Yang W., Parr R.G. (1988). Development of the Colle-Salvetti Correlation-Energy Formula into a Functional of the Electron Density. Phys. Rev. B.

[65] McLean A.D., Chandler G.S. (1980). Contracted Gaussian basis sets for molecular calculations. I. Second row atoms, Z=11-18. J. Chem. Phys..

[66] Krishnan R., Binkley J.S., Seeger R., Pople J.A. (1980). Self-consistent molecular orbital methods. XX. A basis set for correlated wave functions. J. Chem. Phys..

[67] Frisch M.J., Pople J.A., Binkley J.S. (1984). Self-consistent molecular orbital methods 25. Supplementary functions for Gaussian basis sets. J. Chem. Phys..

[68] Frisch M.J., Trucks G.W., Schlegel H.B., Scuseria G.E., Robb M.A., Cheeseman J.R., Scalmani G., Barone V., Petersson G.A., Nakatsuji H. (2016). Gaussian 16, Revision C.01.

[69] Schaftenaar G., Noordik J.H. (2000). Molden: A pre- and post-processing program for molecular and electronic structures. J. Comput. Aided Mol. Des..

[70] Martin J.M.L., Van Alsenoy C. (1995). GAR2PED.

[71] Dovesi R., Orlando R., Civalleri B., Roetti C., Saunders V.R., Zicovich-Wilson C.M. (2005). CRYSTAL: A computational tool for the *ab initio* study of the electronic properties of crystals. Z. Kristallogr..

[72] Dovesi R., Saunders V.R., Roetti C., Orlando R., Zicovich-Wilson C.M., Pascale F., Civalleri B., Doll K., Harrison N.M., Bush I.J. (2009). CRYSTAL09 User’s Manual.

[73] Grimme S. (2006). Semiempirical GGA-type density functional constructed with a long-range dispersion correction. J. Comput. Chem..

[74] Surov A.O., Voronin A.P., Vener M.V., Churakov A.V., Perlovich G.L. (2018). Specific features of supramolecular organisation and hydrogen bonding in proline cocrystals: A case study of fenamates and diclofenac. CrystEngComm.

[75] Voronin A.P., Surov A.O., Churakov A.V., Parashchuk O.D., Rykounov A.A., Vener M.V. (2020). Combined X-ray Crystallographic, IR/Raman Spectroscopic, and Periodic DFT Investigations of New Multicomponent Crystalline Forms of Anthelmintic Drugs: A Case Study of Carbendazim Maleate. Molecules.

[76] Surov A.O., Vasilev N.A., Churakov A.V., Parashchuk O.D., Artobolevskii S.V., Alatortsev O.A., Makhrov D.E., Vener M.V. (2021). Two Faces of Water in the Formation and Stabilization of Multicomponent Crystals of Zwitterionic Drug-Like Compounds. Symmetry.

[77] Vener M.V., Sauer J. (2005). Environmental effects on vibrational proton dynamics in H_5_O_2_^+^: DFT study on crystalline H_5_O_2_^+^ClO_4_^−^. Phys. Chem. Chem. Phys..

[78] Sen A., Mitev P.D., Eriksson A., Hermansson K. (2016). H-bond and electric field correlations for water in highly hydrated crystals. Int. J. Quantum Chem..

[79] Červinka C., Fulem M. (2019). Cohesive properties of the crystalline phases of twenty proteinogenic α-aminoacids from first-principles calculations. Phys. Chem. Chem. Phys..

[80] Beran G.J.O. (2016). Modeling Polymorphic Molecular Crystals with Electronic Structure Theory. Chem. Rev..

[81] Surov A.O., Manin A.N., Voronin A.P., Churakov A.V., Perlovich G.L., Vener M.V. (2017). Weak Interactions Cause Packing Polymorphism in Pharmaceutical Two-Component crystals. The case study of the Salicylamide Cocrystal. Cryst. Growth Des..

