# Supplementary material for: Spectroscopic Identification of Hydrogen Bond Vibrations and Quasi-Isostructural Polymorphism in N-Salicylideneaniline
Source: Molecules. 2021 Aug 20;26(16):5043. doi: 10.3390/molecules26165043 (PMC8401763; doi:10.3390/molecules26165043)
Supplement: Supplementary file 1 [file molecules-26-05043-s001.zip › molecules-1343615-supplementary.pdf]

Supplementary material.

Spectroscopic identification of hydrogen bond vibrations and quasi-isostructural polymorphism in N-salicylideneaniline

Łukasz Hetmańczyk,[1] Eugene A. Goremychkin,[2] Janusz Waliszewski,[3] Mikhail Vener,[4, 5] Paweł Lipkowski,[6] Peter M. Tolstoy[7, *] and Aleksander Filarowski[2, 8 *]

Faculty of Chemistry, Jagiellonian University, 2 Gronostajowa str., 30-387 Cracow, Poland; lukasz.hetmanczyk@uj.edu.pl

2 Frank Laboratory of Neutron Physics, Joint Institute for Nuclear Research 6 F. Joliot-Curie str., 141980 Dubna, Russia; goremychkin@jinr.ru

3 Faculty of Physics, University of Bialystok, 1L Ciolkowskiego str. 15-245 Bialystok, Poland; j.waliszewski@uwb.edu.pl

4 Quantum Chemistry Department, Mendeleev University of Chemical Technology, Miusskaya Square 9, 125047 Moscow, Russia. venermv@muctr.ru

5 Kurnakov Institute of General and Inorganic Chemistry, Russian Academy of Sciences, Leninskii prosp. 31, 119991 Moscow, Russia. mikhail.vener@gmail.com

6 Department of Physical and Quantum Chemistry, Wrocław University of Science and Technology, Wybrzeże Wyspiańnskiego 27, PL-50370 Wrocław, Poland; pawel.lipkowski@pwr.edu.pl

7 Institute of Chemistry, St. Petersburg State University, Universitetskij pr. 26, 198504 St. Petersburg, Russia; peter.tolstoy@spbu.ru

8 Faculty of Chemistry, University of Wrocław 14 F. Joliot-Curie str., 50-383 Wrocław, Poland; aleksander.filarowski@chem.uni.wroc.pl

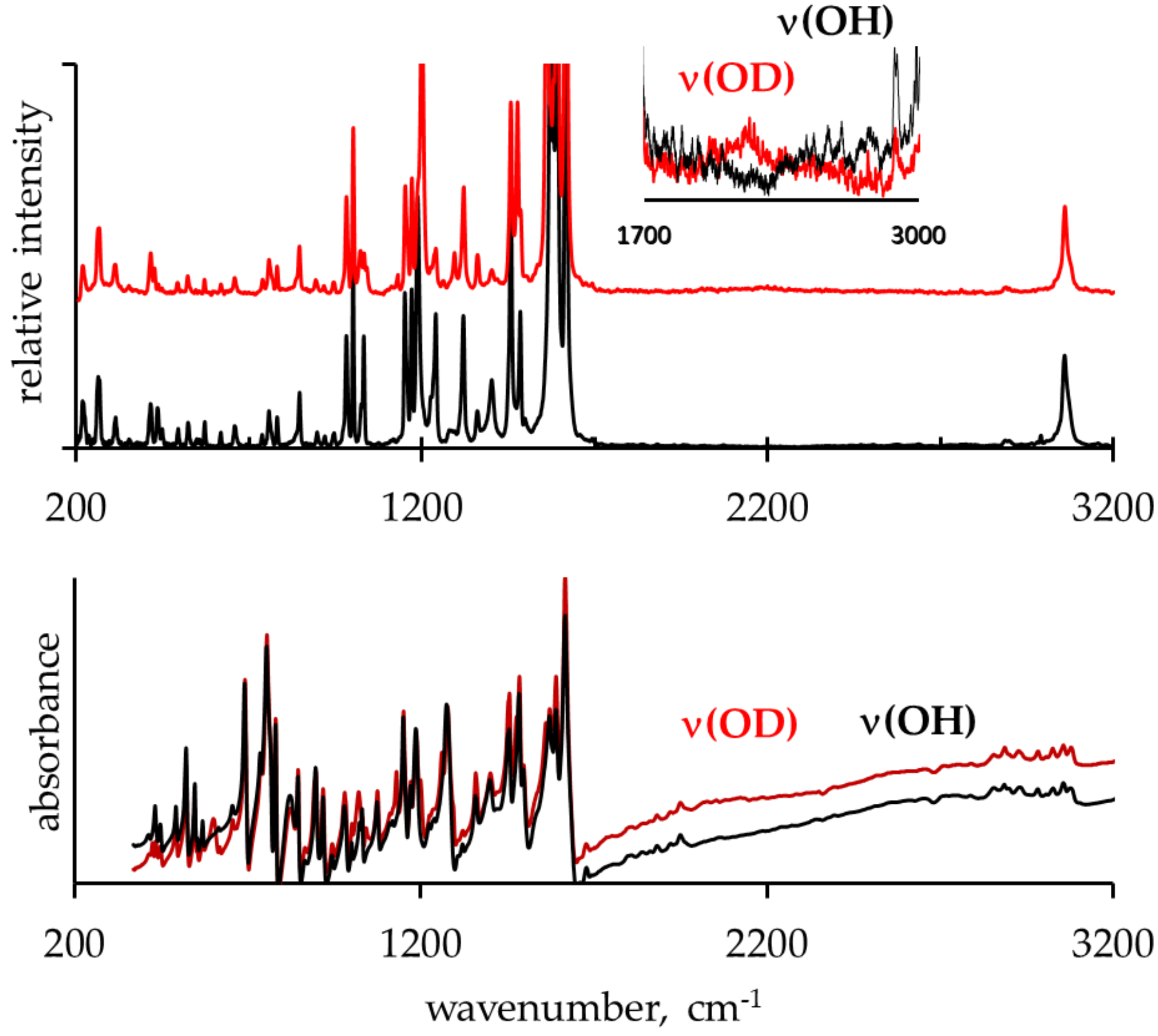

Figure S1. Normalized experimental Raman (**A**) and IR (**B**) spectra of **SA** (black trace) and its deuterated derivative **SA-OD** (red trace).

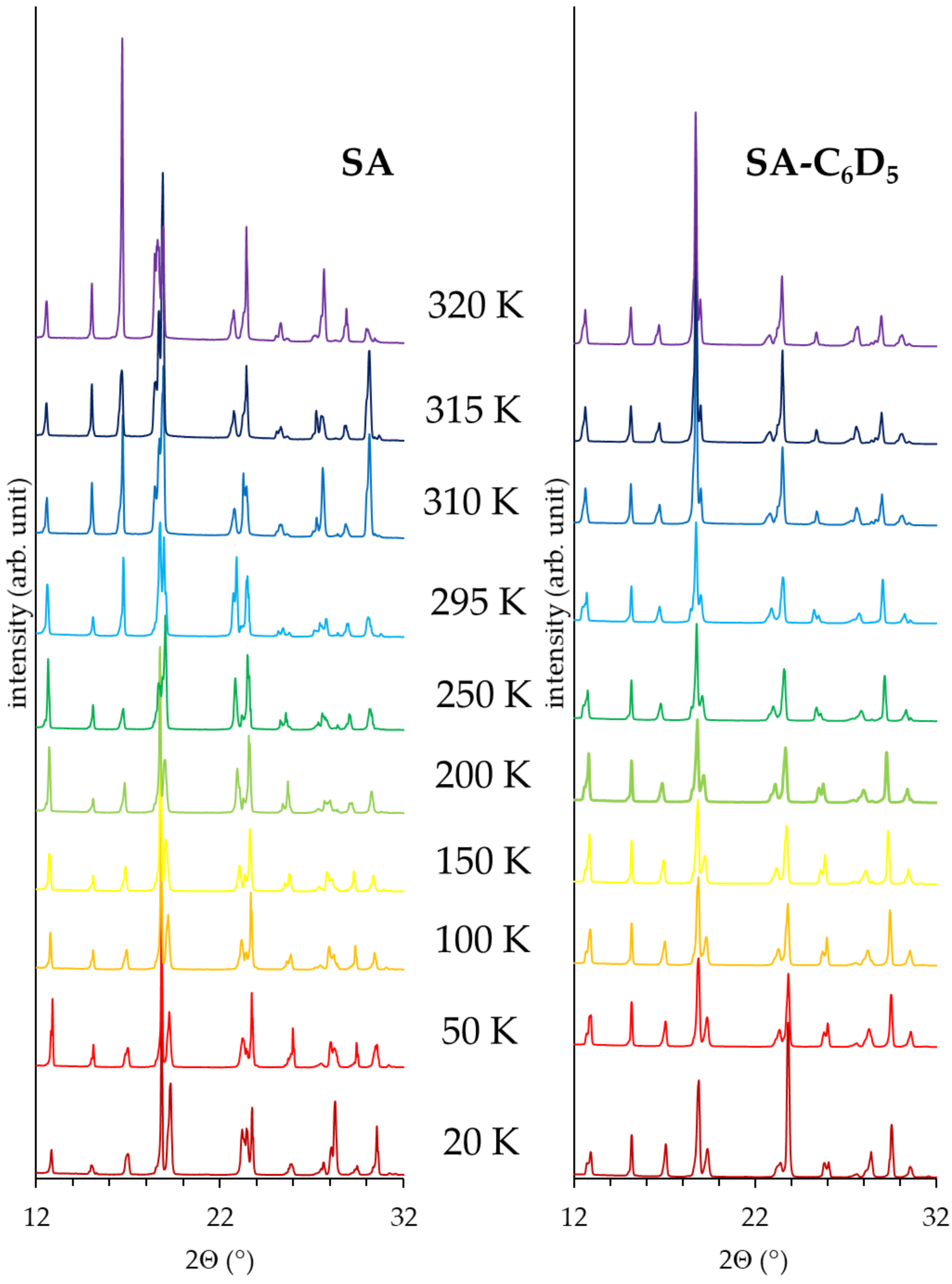

Figure S2. Temperature dependence of X-ray powder diffraction patterns of **SA** and **SA-C_6D_5**.

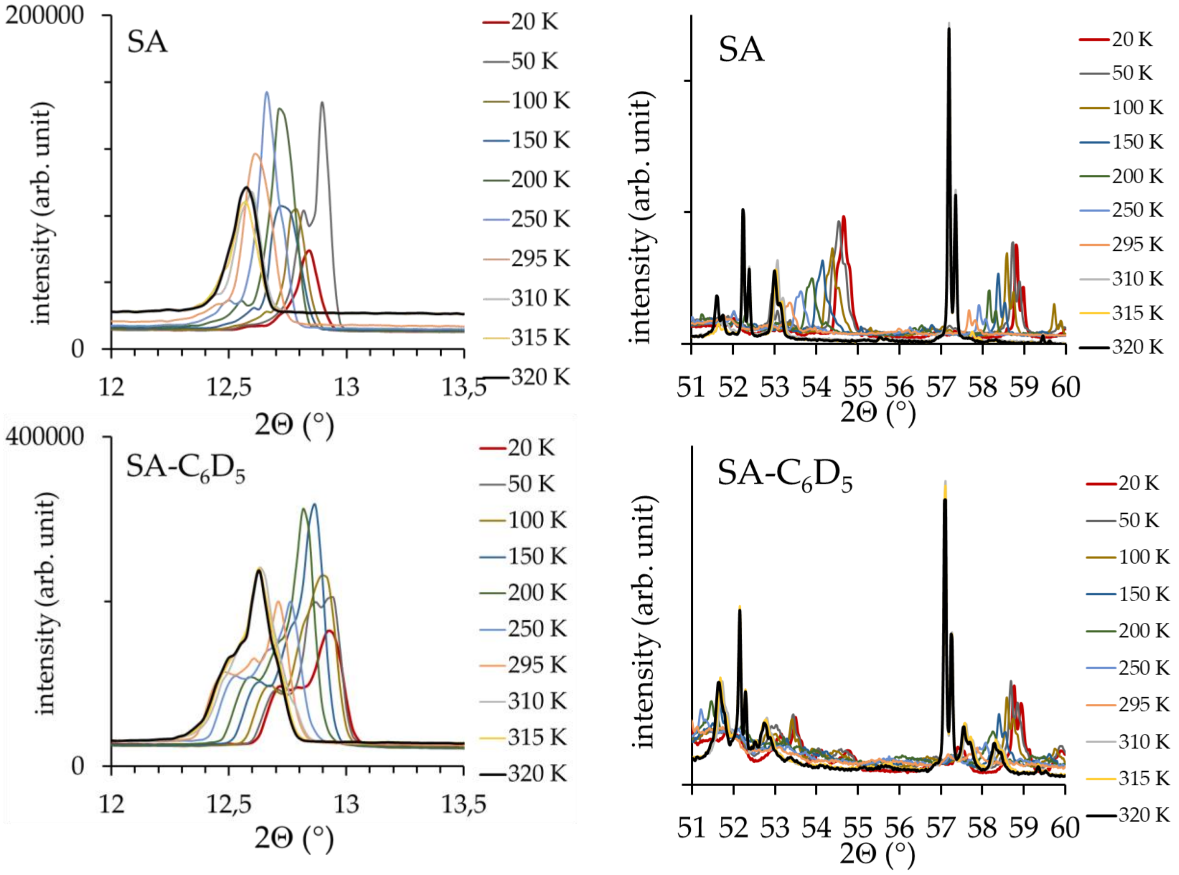

Figure S3. Temperature dependence of X-ray powder diffraction patterns of **SA** and **SA-C_6D_5**.

Table S1. Definitions of the internal coordinates used in the potential energy distribution (PED) analysis for the assignments of the vibrational spectra.

$\nu(C_{ar}H)$ – stretching of the $C_{aryl}H$ bond
$\nu(C_{alk}H)$ – stretching of the $C_{alkyl}H$ bond
ν(OH) – stretching of the O-H bond
ν(OD) – stretching of the O-D bond
ν(CO) – stretching of the $C_{aryl}O$ bond
$\nu(C_{ar}C_{alk})$ – stretching of the $C_{aryl}C_{alkyl}$ bond
$\nu(C_{alk}C_{alk})$ – stretching of the $C_{alkyl}C_{alkyl}$ bond
ν(C=N) – stretching of the C_{aryl}=N bond
ν(C-N) – stretching of the C_{aryl}-N bond
α(CC) – in-plane bending of the $C_{aryl}C_{aryl}C_{aryl}$ chain
$\delta(CC_{alk})$ – in-plane bending of the C(aryl)C(aryl)C(alkyl) chain
δ(CH) – in-plane bending of the C(aryl)C(aryl)H chain
δ(OH) – in-plane bending of the C(aryl)-O-H chain
δ(OD) – in-plane bending of the C(aryl)-O-D chain
δ(CO) – in-plane bending of the C(aryl)=C(aryl)-O chain
δ(C=N) – in-plane bending of the -C(alkyl)=N unit
δ(C-N) – in-plane bending of the N-C(aryl) unit
γ(OH) – out-of-plane bending of the O-H bond (the change in angle between the O-H and C(aryl)-O bonds)
γ(OD) – out-of-plane bending of the O-D bond (the change in angle between the O-H and C(aryl)-O bonds)
γ(C-N) – out-of-plane bending of the C-N bond (the change in angle between the C(aryl)-N and C(aryl)=C(aryl) bonds)
γ(C=N) – out-of-plane bending of the C=N bond (the change in angle between the C(alkyl)-N and C(aryl)=C(aryl) bonds)
$\gamma(CC_{alk})$ – out-of-plane bending of the C(aryl)-C(alkyl) (the change in angle between the C(aryl)-C(alkyl) bond and plane defined by three carbon atoms in the phenyl ring)
γ(CH) – out-of-plane bending of the C(aryl)-H (the change in angle between the C(aryl)-H bond and plane defined by three carbon atoms in the phenyl ring)
γ(CO) – out-of-plane bending of the C(aryl)-O (the change in angle between the C(aryl)-O bond and plane defined by three carbon atoms in the phenyl ring)
τ(CC) – torsion angle in the ring (change in the dihedral angle between two C(aryl)=C(aryl)=C(aryl) planes in the phenyl ring)
τ(CH) – torsion around the C(alkyl)-C(alkyl)-H bond
$\tau(CC_{alk})$ – torsion angle in the ring (change in the dihedral angle between C(aryl)=C(aryl)=C(aryl) and C(aryl)=C(aryl)-C(alkyl) planes)
$\tau(C_{alk}N)$ – torsion angle (change in the dihedral angle between C(aryl)-C(alkyl)-C(aryl) and C(aryl)-C(alkyl)=N planes)
$\tau(CC_{alk})$ – torsion angle (change in the dihedral angle between C(aryl)=C(aryl)=C(alkyl) and C(aryl)=C(aryl)=C(alkyl) planes)
τ(N-C) – torsion angle (change in the dihedral angle between C(aryl)=C(aryl)=C(aryl) and C(aryl)=C(aryl)-N planes)

Table S2. Experimental IR, Raman, IINS and calculated (B3LYP/6-311+G(d,p)) spectral data of **SA** compound.

№	IR(**SA**), cm^{-1}	R(**SA**) cm^{-1}	IINS (**SA**), cm^{-1}	DFT, [cm^{-1}]	Int. [km/mol]	Potential energy distribution (%)
1	2600-2000			3213.7	512.50	ν(OH) (99)
2	3077			3196.4	7.46	ν(C$_{ar}$H) (97)
3				3192.2	16.35	ν(C$_{ar}$H) (95)
4	3054	3057		3189.6	14.97	ν(C$_{ar}$H) (94)
5	3027			3184.7	21.69	ν(C$_{ar}$H) (100)
6	3003sm			3178.3	11.40	ν(C$_{ar}$H) (94)
7	2982	2989		3168.3	2.69	ν(C$_{ar}$H) (96)
8	2931			3168.2	10.30	ν(C$_{ar}$H) (99)
9		2897		3160.7	4.62	ν(C$_{ar}$H) (95)
10	2898sm	2891		3155.2	3.49	ν(C$_{ar}$H) (96)
11	2884			3030.4	39.15	ν(C$_{alk}$H) (98)
12	1616	1614		1666.5	235.41	ν(C=N) (41) δ(CH) (12) δ(CC$_{alk}$) (7) ν(C$_{ar}$C$_{alk}$) (7)
13	1590	1590		1662.2	28.96	ν(C$_{alk}$C$_{alk}$) (42) δ(OH) (11) α(CC) (8) γ(CH) (6) ν(C=N) (6)
14		1580sm		1631.2	110.52	ν(C$_{alk}$C$_{alk}$) (51) α(CC) (7)
15				1614.3	4.50	ν(C$_{alk}$C$_{alk}$) (66) γ(CH) (10) α(CC) (9)
16	1571	1571		1607.4	142.08	ν(C$_{alk}$C$_{alk}$) (32) δ(OH) (13) ν(C=N) (12) γ(CH) (9)
17	1540			1531.8	37.66	δ(OH) (16) γ(CH) (24) ν(C$_{alk}$C$_{alk}$) (20)
18	1533sh			1516.6	54.40	γ(CH) (49) ν(C$_{alk}$C$_{alk}$) (26)
19	1496	1499		1489.8	68.84	ν(C$_{alk}$C$_{alk}$) (33) ν(CO) (10) γ(CH) (23) δ(CC$_{alk}$) (8) ν(C=N) (6)
20	1484	1484		1479.9	3.94	γ(CH) (48) ν(C$_{alk}$C$_{alk}$) (28)
21	1455	1456		1449.2	39.15	δ(OH) (35) γ(CH) (28) ν(C$_{alk}$C$_{alk}$) (19) ν(C$_{ar}$C$_{alk}$) (5)
22	1419					
23	1400	1400		1405.3	19.68	δ(CH) (56) ν(C=N) (9) ν(C$_{ar}$C$_{alk}$) (5) γ(CH) (5)
24				1355.4	0.01	ν(C$_{alk}$C$_{alk}$) (48) δ(C=N) (6) γ(CH) (12)
25	1358	1359		1351.8	4.70	γ(CH) (36) ν(C$_{alk}$C$_{alk}$) (26)
26	1320s	1318		1320.1	8.53	ν(C$_{alk}$C$_{alk}$) (68) γ(CH) (12) ν(CO) (5)
27	1306s	1294s		1315.8	63.40	ν(CO) (30) α(CC) (13) γ(CH) (9) ν(C$_{ar}$C$_{alk}$) (6)
28		1284s				
29	1276			1268.2	5.05	ν(C$_{alk}$C$_{alk}$) (16) γ(CH) (15) ν(CO) (9) δ(CH) (9) ν(C-N) (9) ν(C$_{ar}$C$_{alk}$) (9) δ(C=N) (7) δ(CC$_{alk}$) (6)
30	1239	1238		1254.7	14.49	ν(C$_{alk}$C$_{alk}$) (17) γ(CH) (30) ν(C-N) (14) δ(CO) (6)
31	1224	1225s		1214.7	74.79	ν(C-N) (15) ν(C$_{ar}$C$_{alk}$) (11) γ(CH) (22) ν(C$_{alk}$C$_{alk}$) (19)
32				1200.7	23.10	γ(CH) (55) ν(C-N) (10)

33	1186	1187	1185	1189.4	0.62	γ(CH) (75) ν(C$_{alk}$C$_{alk}$) (15)
34	1171	1169	1175	1186.1	42.45	γ(CH) (61) ν(C$_{alk}$C$_{alk}$) (8)
35	1149	1150	1153	1141.5	9.05	γ(CH) (35) ν(C$_{alk}$C$_{alk}$) (24) δ(CC$_{alk}$) (8) α(CC) (8) δ(CO) (5)
36	1118	1118s				
37	1110		1089	1107.7	6.92	ν(C$_{alk}$C$_{alk}$) (49) γ(CH) (37)
38	1074		1087	1053.3	6.92	ν(C$_{alk}$C$_{alk}$) (64) γ(CH) (18)
39	1031	1032		1046.7	5.87	ν(C$_{alk}$C$_{alk}$) (53) α(CC) (7) γ(CH) (13)
40	1023	1024s		1015.1	1.32	α(CC) (50) γ(C=N) (17) ν(C$_{alk}$C$_{alk}$) (11)
41				1002.9	9.51	γ(C=N) (65) α(CC) (6) γ(CC$_{alk}$) (6) τ(CC$_{alk}$) (5)
42	1000	1000		998.5	0.11	τ(CH) (79) τ(CC) (15)
43			992v.b.	986.6	0.29	τ(CH) (80) τ(CC) (12)
44	980	980		980.6	0.11	τ(CH) (89) τ(CC) (8)
45				952.9	1.25	τ(CH) (84) τ(CC) (7)
46	943	944		932.7	12.95	τ(CH) (47) δ(C=N) (15) α(CC) (14) τ(CC) (5)
47	918	919	919	910.1	19.73	τ(CH) (42) α(CC) (19) δ(C=N) (16)
48	897	895		872.3	2.92	τ(CH) (56) γ(OH) (12) τ(CC) (10) γ(CO) (8)
49	865			860.4	12.87	α(CC) (32) ν(C-N) (14) δ(C=N) (11) ν(C$_{alk}$C$_{alk}$) (16)
50			855	850.8	65.09	γ(OH) (66) τ(CH) (15) γ(CO) (6)
51	846	843		843.4	0.66	τ(CH) (98)
52	825			796.8	20.78	ν(C$_{alk}$C$_{alk}$) (27) τ(CH) (17) τ(CC) (8) ν(CO) (8) α(CC) (6)
53	781	780	777	771.7	38.09	τ(CH) (36) τ(CC) (24) ν(C$_{alk}$C$_{alk}$) (5)
54	755	755		763.6	63.45	τ(CH) (71) γ(CO) (15) τ(CC) (8)
55	737	736		744.8	2.52	τ(CC) (49) γ(CC$_{alk}$) (18) γ(CO) (17) τ(CH) (6)
56	691			704.9	36.06	τ(CC) (60) τ(CH) (36)
57	657	657	658	672.2	5.49	α(CC) (51) δ(C=N) (23)
58	617s	615	625	631.9	0.02	α(CC) (84)
59	571	570		583.8	5.29	α(CC) (63) δ(CC$_{alk}$) (9)
60	555w	554s	557	563.0	1.93	τ(CC) (54) γ(CO) (9) γ(CC$_{alk}$) (8) τ(CH) (5)
61	548	551s		556.2	11.77	δ(CC$_{alk}$) (19) α(CC) (20) δ(CO) (9)
62	523	522	538	533.9	14.79	τ(CC) (30) τ(CH) (22) α(CC) (14) γ(CO) (8.)
63	494	493	497	501.4	2.54	δ(C-N) (16) τ(CC) (12) γ(CO) (12) γ(CC$_{alk}$) (9) δ(CC$_{alk}$) (8) δ(C=N) (6)
64				460.4	3.16	δ(CO) (25) γ(CC$_{alk}$) (17) τ(CC) (28) α(CC) (6)
65	449	446	445	447.4	3.83	τ(CC) (31) γ(CC$_{alk}$) (20) δ(CO) (16) δ(C=N) (7)
66	434	434				

67	416	416	416	420.6	0.93	τ(CC) (82)
68	353		359	347.3	0.68	τ(CC) (49) δ(CC$_{alk}$) (13) δ(C-N) (5)
69	311	312	313	318.5	3.58	τ(CC$_{alk}$) (23) γ(CC$_{alk}$) (12) δ(C-N) (10) δ(CC$_{alk}$) (9) γ(CO) (7) γ(OH) (7) τ(N-C) (7)
74	261	264	264	264.6	0.94	τ(CC) (31) τ(CC$_{alk}$) (7) δ(CC$_{alk}$) (7) α(CC) (7) ν(C$_{ar}$C$_{alk}$) (6)
75	222	217	222	219.5	1.31	τ(CC) (45) τ(CC$_{alk}$) (21) τ(C$_{alk}$N) (8) γ(CC$_{alk}$) (7) τ(N-C) (7)
76			208			
77	202		165	199.2	1.48	δ(CC$_{alk}$) (16) δ(C=N) (15) τ(CC) (26) δ(C-N) (6) τ(C$_{alk}$N) (5)
78	158		124	147.0	0.34	γ(OH) (24) τ(CC) (17)- τ(C$_{alk}$N) (14) γ(CC$_{alk}$) (8) τ(N-C) (6) τ(CC$_{alk}$) (5)
79	106		88	76.6	0.15	δ(C=N) (34) τ(CC$_{alk}$) (10) δ(CC$_{alk}$) (10) τ(CH) (8) δ(C-N) (7) γ(OH) (6) τ(CC) (5)
80	59.3		51	55.9	0.17	γ(CC$_{alk}$) (41) τ(C$_{alk}$N) (14) γ(OH) (10) γ(C=N) (10) τ(CH) (8)
81			39	41.0	0.54	τ(N-C) (57) τ(CC$_{alk}$) (19) δ(C-N) (10)

ν - stretching vibration; δ - scissoring vibration; ρ - rocking; ω - wagging; γ – out-of-plane; τ - twisting and torsional vibtration; sh - shoulder and s – small intensity.

Table S3. Experimental IR, Raman and calculated (B3LYP/6-311+G(d,p)) spectral data of deuterated derivative **SA-OD**.

	IR(**SA-OD**), cm^{-1}	R(**SA-OD**), cm^{-1}	DFT, cm^{-1}	Int. [km/mol]	Potential energy distribution (%)
1			3196.4	10.79	ν(C_{ar}H) (97)
2			3192.2	13.84	ν(C_{ar}H) (95)
3			3189.6	13.72	ν(C_{ar}H) (95)
4	3079		3184.8	17.41	ν(C_{ar}H) (100)
5	3055	3059	3178.3	11.10	ν(C_{ar}H) (95)
6	3023		3168.3	2.05	ν(C_{ar}H) (90)
7	3004		3168.3	6.40	ν(C_{ar}H) (95)
8	2981		3160.7	3.74	ν(C_{ar}H) (95)
9	2927		3155.3	4.89	ν(C_{ar}H) (96)
10	2884		3030.9	29.06	ν(C_{alk}H) (98)
11	2400-1700		2344.4	296.94	ν(OD) (99)
12	1677		1666.2	229.88	ν(C=N) (41) δ(CH) (12) δ(CC_{alk}) (7) ν($C_{ar}C_{alk}$) (7)
13	1616	1616	1651.5	17.05	ν($C_{alk}C_{alk}$) (57) α(CC) (9) ν(C=N) (8) γ(CH) (8)
14	1590	1590	1629.9	121.47	ν($C_{alk}C_{alk}$) (51) α(CC) (8) ν(C=N) (6) γ(CH) (10)
15	1571	1580sh	1614.3	4.20	ν($C_{alk}C_{alk}$) (65) γ(CH) (10) α(CC) (9)
16	1560	1561	1588.5	125.59	ν($C_{alk}C_{alk}$) (51) γ(CH) (12) ν(C=N) (7) α(CC) (6)
17	1532sh		1521.0	31.82	γ(CH) (52) ν($C_{alk}C_{alk}$) (31) ν(C-N) (6)
18	1496		1504.2	74.47	γ(CH) (41) ν($C_{alk}C_{alk}$) (27) ν($C_{ar}C_{alk}$) (8)
19	1484	1484	1486.4	44.32	γ(CH) (27) ν($C_{alk}C_{alk}$) (20) ν(CO) (8) δ(CC_{alk}) (7) ν(C=N) (6)
20	1475sm	1476	1479.6	4.21	γ(CH) (43) ν($C_{alk}C_{alk}$) (25)
21	1455	1456			
22	1436sh				
23	1419sm				
24	1400	1402	1405.4	17.35	δ(CH) (56) ν(C=N) (9) ν($C_{ar}C_{alk}$) (6)
25	1385sh		1360.1	2.50	ν($C_{alk}C_{alk}$) (72)
26	1358	1360	1352.3	4.72	γ(CH) (53) ν($C_{alk}C_{alk}$) (28)
27	1321	1320	1323.2	27.15	ν(CO) (18) γ(CH) (34) α(CC) (6)
28	1306sm		1319.9	13.41	ν($C_{alk}C_{alk}$) (65) ν(CO) (8) γ(CH) (12)
29	1294sh	1294			
30	1276		1290.7	16.09	γ(CH) (21) ν(CO) (15) δ(C=N) (11) δ(CC_{alk}) (10) δ(COD) (9) ν($C_{alk}C_{alk}$) (9) α(CC) (8.)
31	1260	1262ws	1264.3	6.77	ν(C=N) (18) ν($C_{ar}C_{alk}$) (18) δ(CH) (10) ν($C_{alk}C_{alk}$) (17) γ(CH) (5)
32	1235	1239			

33	1224sm		1222.9	92.57	ν(C=N) (23) ν($C_{ar}C_{alk}$) (11) ν($C_{alk}C_{alk}$) (9) α(CC) (7) γ(CH) (11)
34	1198sm	1199	1202.1	11.10	γ(CH) (67) ν(C=N) (6) ν($C_{alk}C_{alk}$) (6)
35	1186	1187sh	1189.5	0.74	γ(CH) (77) ν($C_{alk}C_{alk}$) (16)
36	1170	1171	1186.8	26.94	γ(CH) (74) ν($C_{alk}C_{alk}$) (13)
37	1150	1151	1158.5	31.85	γ(CH) (46) ν($C_{alk}C_{alk}$) (24) δ(COD) (10) δ(CC_{alk}) (5)
38	1129	1132ws			δ(COD)
39	1119sh				
40	1111sm		1107.7	7.73	ν($C_{alk}C_{alk}$) (49) γ(CH) (37)
41	1094sh				
42	1074				
43			1065.8	10.01	δ(COD) (35) ν($C_{alk}C_{alk}$) (33) γ(CH) (8)
44	1041	1040w	1047.5	8.56	ν($C_{alk}C_{alk}$) (50) γ(CH) (13)
45	1031sm	1032	1041.2	25.94	δ(COD) (33) ν($C_{alk}C_{alk}$) (33) α(CC) (8)
46	1020	1020	1015.1	1.14	α(CC) (50) γ(C=N) (17) ν($C_{alk}C_{alk}$) (12)
47	1001	1001	1002.8	8.59	γ(C=N) (65) α(CC) (6) γ(CC_{alk}) (6) τ(CC_{alk}) (5.)
48			998.5	0.10	τ(CH) (79) τ(CC) (15)
49	979		986.6	0.32	τ(CH) (80) τ(CC) (12)
50		981	980.6	0.11	τ(CH) (89) τ(CC) (8)
51	965sh				
52	943	943w	952.8	1.53	τ(CH) (84) τ(CC) (7)
53			930.6	11.76	τ(CH) (58) δ(C=N) (11) α(CC) (8) τ(CC) (6)
54	917	916w	905.0	20.77	δ(C=N) (21) α(CC) (21) τ(CH) (33)
55	896	892w			
56			868.3	3.52	τ(CH) (66) τ(CC) (12) γ(CO) (11) γ(CC_{alk}) (5)
57	865		859.9	12.22	α(CC) (32) ν($C_{alk}C_{alk}$) (16) ν(C-N) (14) δ(C=N) (10)
58	845	845w	843.3	0.86	τ(CH) (99)
59	823				
60	780	779w	795.8	19.91	ν($C_{alk}C_{alk}$) (26) τ(CH) (17) τ(CC) (8) ν(CO) (8) α(CC) (6)
61	767sh		771.5	39.14	τ(CC) (24) τ(CH) (36) ν($C_{alk}C_{alk}$) (5)
62	755	759w	763.7	57.20	τ(CH) (74) γ(CO) (14) τ(CC) (7)
63			746.1	4.02	τ(CC) (48) γ(CC_{alk}) (18) γ(CO) (18) τ(CH) (5)
64	737	739w			
65	720sh				
66	692		704.9	37.42	τ(CC) (60.) τ(CH) (36)

67	669sh		671.7	5.86	α(CC) (52) δ(C=N) (24)
68	656				
69		657w			
70			632.7	3.14	α(CC) (75) γ(COD) (8)
71	618sm	618w	624.5	30.70	γ(COD) (79) α(CC) (8) τ(CC) (5)
72	601				
73			581.9	5.90	α(CC) (68) δ(CC_{alk}) (7)
74	570	570w			
75			561.8	1.91	τ(CC) (32) α(CC) (22) γ(CC_{alk}) (8) γ(CO) (8) τ(CH) (5)
76			553.2	12.22	δ(CC_{alk}) (20) α(CC) (16) δ(CO) (6) τ(CC) (6) δ(C=N) (5) τ(CH) (5)
77	554sm				
78	547		531.9	16.78	τ(CC) (22) τ(CH) (15) α(CC) (23) γ(CO) (7)
79	522	520w			
80	491	493w	499.2	4.91	δ(C-N) (15) τ(CC) (14) γ(CO) (13.) γ(CC_{alk}) (9) δ(CC_{alk}) (7) δ(C=N) (5)
81	479sh				
82	448		455.9	2.40	γ(CC_{alk}) (26) τ(CC) (43) δ(CO) (6) τ(CH) (5) γ(CO) (5)
83	434		436.4	4.28	δ(CO) (43) τ(CC) (11) δ(C=N) (11) γ(CC_{alk}) (8)
84	425	426w	420.4	0.90	τ(CC) (81)
85	415sm	414w			
86		348w	345.8	0.92	τ(CC) (50) δ(CC_{alk}) (12)
87		310w	317.5	3.15	τ(CC_{alk}) (23) γ(CC_{alk}) (12) δ(C-N) (10) δ(CC_{alk}) (10) γ(OH) (8) γ(CO) (7) τ(N-C) (7)
88		266w	264.2	0.89	τ(CC) (31) δ(CC_{alk}) (7) τ(CC_{alk}) (7) α(CC) (7) ν($C_{ar}C_{alk}$) (7)
89		217w	217.3	1.13	τ(CC) (44) τ(CC_{alk}) (22) τ($C_{alk}N$) (8) γ(CC_{alk}) (7) τ(N-C) (7)
90			197.6	1.49	δ(CC_{alk}) (16) δ(C=N) (15) τ(CC) (26) δ(C-N) (6) τ($C_{alk}N$) (5)
91			146.8	0.35	γ(OH) (23) τ(CC) (17) τ($C_{alk}N$) (14) γ(CC_{alk}) (8) τ(N-C) (6) τ(CC_{alk}) (5)
92			76.3	0.15	δ(C=N) (34) τ(CC_{alk}) (10) δ(CC_{alk}) (10) τ(CH) (8) δ(C-N) (6) γ(COD) (6) τ(CC) (5)
93			55.8	0.16	γ(CC_{alk}) (41) τ($C_{alk}N$) (14.) γ(OH) (10) γ(C=N) (10) τ(CH) (8)
94			40.9	0.53	τ(N-C) (57) τ(CC_{alk}) (19) δ(C-N) (10)

Table S4. Experimental IINS and calculated (B3LYP/6-311+G(d,p)) spectral data of deuterated **SA-C_6D_5** derivative for 0 – 1200 region.

	IINS (**SA-C_6D_5**), cm^{-1}	DFT, cm^{-1}	Int. [km/mol]	Potential energy distribution (%)
1	1163	1186.68	16.90	γ(CH) (76) ν($C_{alk}C_{alk}$) (8)
2	-	1175.85	92.67	ν(C-N) (35) α(CC) (13) ν($C_{alk}C_{alk}$) (5)
3	-	1141.11	6.87	ν($C_{alk}C_{alk}$) (28) γ(CH) (37) α(CC) (8) δ_{alk}(CC_{alk}) (6) δ(CO) (5)
4	-	1056.88	1.44	γ(CD) (80) δ(C-N) (6) ν($C_{alk}C_{alk}$) (5)
5	-	1052.34	4.55	ν($C_{alk}C_{alk}$) (59) γ(CH) (15)
6	-	1005.52	10.40	γ(C=N) (70) τ(CC=N) (9) τ(CH) (6) γ(CC_{alk}) (5)
7	990	986.59	0.27	τ(CH) (80) τ(CC) (12)
8	-	970.17	1.06	α(CC) (58) ν($C_{alk}C_{alk}$) (36)
9	-	952.68	0.95	τ(CH) (86) τ(CC) (7)
10	-	920.04	19.85	α(CC) (39) δ(C=N) (20)
11	-	889.64	0.94	γ(CD) (46) ν($C_{alk}C_{alk}$) (18) α(CC) (14)
12	-	872.69	3.77	τ(CH) (56) γ(OH) (12) τ(CC) (10) γ(CO) (8)
13	-	863.05	2.13	γ(CD) (54) α(CC) (13) ν($C_{alk}C_{alk}$) (13) ν(C-N) (5)
14	-	860.53	1.48	γ(CD) (81) ν($C_{alk}C_{alk}$) (6)
15	855	851.37	57.58	γ(OH) (63) τ(CH) (15) γ(CO) (6)
16	-	839.92	10.57	τ(CC) (25) γ(CD) (30) τ(CD) (13) γ(CN) (7)
17	-	836.17	4.17	τ(CC) (39) τ(CD) (38) γ(CN) (8) γ(CD) (6)
18	-	815.60	11.36	τ(CD) (31) τ(CC) (14) ν($C_{alk}C_{alk}$) (7) γ(CD) (5)
19	-	797.10	0.41	τ(CD) (82) τ(CC) (18)
20	-	782.32	11.25	γ(CN) (12) τ(CD) (15) τ(CC) (9) γ(CD) (6) ν($C_{alk}C_{alk}$) (5)
21	-	775.65	3.85	γ(CN) (17) τ(CD) (27) τ(CC) (12) ν($C_{alk}C_{alk}$) (10)
22	770	763.59	72.02	τ(CH) (74) γ(CO) (15) τ(CC) (7)
23	-	745.03	3.30	τ(CC) (51) γ(CO) (19) γ(CC_{alk}) (14) τ(CH) (6)
24	649	664.90	3.41	α(CC) (49) δ(C=N) (8) τ(CD) (12)
25	-	655.47	0.84	τ(CD) (91)
26	-	645.31	6.19	γ(CN) (23) τ(CD) (35) τ(CC) (17) δ(C=N) (5)
27		606.93	0.10	α(CC) (78) γ(CD) (6)
28		580.84	5.74	α(CC) (64) δ(CC_{alk}) (7)
29		562.14	9.01	τ(CC) (61) γ(CO) (8)
30	550	555.62	9.86	τ(CC) (50) γ(CO) (10)
31	-	544.47	7.41	α(CC) (42) δ(CO) (10) δ(CC_{alk}) (10)

32	491	496.87	7.01	δ(C-N) (16) δ(CC_{alk}) (12) α(CC) (16) δ(C=N) (6) τ(CC_{alk}) (5)
33	-	483.35	25.41	τ(CC) (29) γ(CC_{alk}) (16) γ(CO) (14) γ(CN) (6)
34	432	452.83	2.19	δ(CO) (57) α(CC) (9) δ(C=N) (8) γ(CN) (5)
35		434.44	4.39	τ(CC) (43) γ(CN) (17) γ(CC_{alk}) (13) δ(C-N) (6)
36	361	367.39	0.90	τ(CC) (87)
37	345			
38	314	338.77	0.88	τ(CC) (52) δ(CC_{alk}) (7) γ(CO) (7) τ(CH) (5)
39	255	314.82	3.53	τ(CC=N) (26) δ(C-N) (13) δ(CC_{alk}) (9) γ(CC_{alk}) (7) τ(N-C) (7) γ(OH) (7) τ(CC) (6) γ(CO) (5)
40	224	254.67	0.71	τ(CC) (38) α(CC) (12) τ(CC=N) (8) ν($C_{ar}C_{alk}$) (7)
41	202	218.33	1.26	τ(CC) (47) τ(CC=N) (25) τ(C=N) (7) τ(N-C) (6) γ(CC_{alk}) (5)
42	164	194.02	1.48	τ(CC) (27) δ(CC_{alk}) (14) δ(C=N) (10) τ(CC_{alk}) (9) δ(C-N) (8)
43	124sh	143.25	0.28	γ(OH) (23) τ(C=N) (20) τ(CC) (20) τ(CC=N) (6) γ(CC_{alk}) (6) τ(N-C) (5)
44	90	74.05	0.02	τ(CC_{alk}) (31) δ(C=N) (15) τ(CC=N) (10) γ(CN) (10) δ(C-N) (7) δ(CC_{alk}) (6) τ(CC) (5)
45	51	54.44	0.78	γ(CC_{alk}) (32) γ(CN) (15) τ(C=N) (12) γ(OH) (11) γ(C=N) (9) τ(N-C) (5)
46	38	38.66	0.24	τ(N-C) (51) τ(CC=N) (21) δ(C-N) (10) τ(CC_{alk}) (8)
